# ACT001 inhibited CD133 transcription by targeting and inducing Olig2 ubiquitination degradation

**DOI:** 10.1038/s41389-023-00462-6

**Published:** 2023-03-30

**Authors:** Huiting Deng, Hailin Liu, Guoyue Yang, Dandan Wang, Ying Luo, Chenglong Li, Zhenchang Qi, Zhili Liu, Peng Wang, Yanfang Jia, Yingtang Gao, Yahui Ding

**Affiliations:** 1 Tianjin Key Laboratory of Extracorporeal Life Support for Critical Diseases, Artificial Cell Engineering Technology Research Center, Tianjin Institute of Hepatobiliary Disease, Tianjin Third Central Hospital affiliated to Nankai University, Nankai University, 83 Jintang Road, Tianjin, 300170 People’s Republic of China; 2 State Key Laboratory of Medicinal Chemical Biology, College of Chemistry, Nankai University, Haihe Education Park, 38 Tongyan Road, Tianjin, 300353 People’s Republic of China; 3 Department of Lung Cancer, Tianjin Medical University Cancer Institute and Hospital, Tianjin, 300060 People’s Republic of China

**Keywords:** Chemotherapy, Target validation

## Abstract

Lung cancer is the most lethal malignancies with high aggressive and poor prognosis. Until now, the five-year survival rate has not been improved which brings serious challenge to human health. Lung cancer stem cells (LCSCs) serve as the root of cancer occurrence, progression, recurrence, and drug resistance. Therefore, effective anti-cancer agents and molecular mechanisms which could specifically eliminate LCSCs are urgently needed for drug design. In this article, we discovered Olig2 was overexpressed in clinical lung cancer tissues and acted as a transcription factor to regulate cancer stemness by regulating CD133 gene transcription. The results suggested Olig2 could be a promising target in anti-LCSCs therapy and new drugs targeted Olig2 may exhibit excellent clinical results. Furthermore, we verified ACT001, a guaianolide sesquiterpene lactone in phase II clinical trial with excellent glioma remission, inhibited cancer stemness by directly binding to Olig2 protein, inducing Olig2 ubiquitination degradation and inhibiting CD133 gene transcription. All these results suggested that Olig2 could be an excellent druggable target in anti-LCSCs therapy and lay a foundation for the further application of ACT001 in the treatment of lung cancer in clinical.

## Introduction

Lung cancer, the most lethal malignancies caused by both extrinsic and intrinsic factors, is responsible for ~1 million deaths per year [1–3]. Lung cancer stem cells (LCSCs), a unique cell subpopulation that drives cancer initiation and causes cancer relapse, are the root of lung cancer aggressiveness, metastasis, relapse, and poor prognosis [4] which brings great challenge for lung cancer therapy [5]. However, LCSCs are resistant to chemotherapies and the first-line strategies currently including chemotherapies as well as radiotherapeutics could not eradicate LCSCs completely. Under hypoxia microenvironment and therapy pressure, LCSCs are enriched and differentiated to initiate new tumors [6]. As a result, selectively eliminating LCSCs offers a promising strategy to augment current cancer therapies [7].

A number of surface markers of LCSCs have been identified. CD133, a transmembrane glycoprotein with five transmembrane regions, has been widely used as a specific stemness marker in many types of CSCs [8]. Lung cancer cells with highly expressed CD133 shows stemness characteristic including higher drug efflux, metastasis, and tumorigenicity capacity [9]. In patients with platinum-based chemotherapy, CD133 expression is negatively correlated with progression-free survival [10]. Moreover, CD133 is commonly used as a cell surface marker to isolate and enrich LCSCs [11]. Although its protein function is still unclear, CD133 is considered to be a focus in LSCSs eliminating and targets finding to discover anti- LCSCs agents.

Oligodendrocyte lineage transcription factor 2 (Olig2), a bHLH transcription factor, is first discovered to be expressed in the central nervous system to promote neural progenitor cell proliferation [12]. Current research in cancer reveals Olig2 is also highly expressed in glioma and correlated with glioma cell proliferation in orthotopic patient-derived xenograft models [13]. Furthermore, Olig2 plays multiple functions in glioma cell migration, invasion, gliomagenesis, chemotherapy, and radiotherapy resistance [14, 15]. Especially, Olig2 is overexpressed in glioma stem cells (GSCs) compared with differential glioma cells and promotes GSCs proliferation through directly inhibiting p21^WAF1/CIP^ [16] which suggests Olig2 seems to be an important glioma stem cell biomarker. Moreover, Olig2 is sufficient to reprogram differentiated cells into GSCs when combines with Sox2, Pouf3f2, and Sall2 [13, 16, 17]. Although previous studies revealed Olig2 expression is normally restricted to neural tissues [18], recent findings show that Olig2 is overexpressed in a wide range of cancers including melanoma, non-small lung carcinoma, leukemia, and breast cancers which suggested overexpression of Olig2 might be oncogenic [19]. In melanoma, inhibition of Olig2 induces cell apoptosis by increasing p53 expression and suppresses metastasis by regulating EMT transcription factors [20]. In leukemia, Olig2 seems only oncogenic in thymocytes and collaborative with LMO1 and Notch1 to induce highly penetrant leukemia [19]. However, the expression and function of Olig2 in lung cancer and LSCSs are still unknown.

Here in this article, we demonstrated Olig2 was highly expressed in lung cancer and contributed to lung cancer cell stemness by regulating CD133 transcription. Moreover, we revealed that ACT001, a guaianolide sesquiterpene lactones undergoing phase II clinical trial for GBM therapy in America and China has received American and European orphan drug qualifications for the prominent effect, targeted lung cancer stem cells by binding and inducing Olig2 ubiquitination degradation. These findings laid a foundation for further application of ACT001 in lung cancer therapy.

## Materials and methods

### Cell culture

The human lung cancer cell lines A549 and NCI-H820 were obtained from the Type Culture Collection of the Chinese Academy of Sciences (Shanghai, China). Cells were cultured in DMEM (Gibco, #8120441, USA) supplemented with 10% FBS (BI, #2029091, ISL), 100 U/ml penicillin, and 100 μg/ml streptomycin (Solarbio, #P1400, China). The cells were all cultured in a cell incubator at 37 °C with 5% CO_2_. The human lung cancer cell lines A549 and NCI-H820 have been authenticated by STR and tested for mycoplasma contamination.

### Transfection

The coding sequence for human Olig2 was chemically synthesized (Tsingke, Beijing, China) and cloning it into pCMV-N-Flag expression vector. The CD133 promoter sequences were predicted (https://dbtss.hgc.jp/), synthesized, and cloned into pGL3-basic vector. siRNA targeting Olig2 was purchased from RiboBio (Guangzhou, China). And the siRNA target sequence for Olig2 knockdown was: 5′-GCATGCACGACCTCAACAT-3′. A549 or NCI-H820 cells were seeded into 6-well plates at the concentration of 3 × 10^5^/well and incubated at 37 °C overnight. Lipo2000 (Invitrogen, #11668-019, USA) was used to transfect vectors or siRNA into cells. After transfected for 48 hours, the cells were collected for further analysis.

### ACT001 treatment

ACT001 was provided by Accendatech Co., Ltd. (Tianjin, China). A549 and NCI-H820 cells were seeded into six-well plates at the concentration of 3 × 10^5^/well. After cell adherence, ACT001 was added with the final concentration of 5 μM, 10 μM, 20 μM, and 50 μM and incubated for 24–48 hours.

### Immunohistochemistry assay

The lung cancer specimens were obtained from biobank of Tianjin Third Central Hospital (Tianjin, China). Informed consent was obtained from all patients, and the protocol was approved by the Ethics Committee of Tianjin Third Central Hospital. The specimens were fixed with 4% paraformaldehyde for 24 hours, following with gradient alcohol, transparentized with xylene, embedded in paraffin, and cut into 5-μm sections. Immunohistochemistry was performed in accordance with the manufacturer’s protocol (ZSGB-BIO, #PV-9000, China). Sections were conventionally dewaxed and hydrated with gradient ethanol. After antigen retrieval by citric acid buffer (pH 6.0) with microwave, endogenous peroxidase was inactivated with endogenous peroxidase blocker for 10 mins. The sections were blocked and then incubated with primary antibody (CD133, 1:200 and Olig2, 1:100) at 4 °C overnight. After the incubation with reaction enhancer and enzyme conjugated sheep anti-rabbit IgG, the sections were stained with DAB (ZSGB-BIO, #ZLI-9017, China) and redyed with hematoxylin. The digital images (×20 magnification) of the slides were taken with a microscope (BX51, OLYMPUS, Japan). With regard to CD133 and Olig2 staining, the scoring criteria were as follows: positive stained area <5%, 0 point; 5%–25%, 1 point; 26%–50%, 2 points; 51%–75%, 3 points; å 75%, 4 points, and color intensity no staining, 0 points; yellow, 1 point; brown, 2 points; tan, 3 points. The immunostaining score was obtained according to the multiplication of color intensity and positive stained area scores. Simultaneously, IHC staining intensity was scored by two independent blinded pathologists.

### Western blot assay

The cells and tissue samples were lysed in RIPA buffer (Solarbio, #R0020, China) supplemented with protease inhibitors. Equal amounts of protein were separated with 10% SDS polyacrylamide gel (YEASEN, #20325ES62, China) and then transferred to PVDF membranes (Millipore, #IPVH00010, USA). After blocked with 5% skim milk at room temperature for 1 hour, the membranes were subject to relevant primary antibodies specific for Olig2 (1:1000, Affinity #DF8004, USA), CD133 (1:2000, Affinity, #AF5120, USA), Nanog (1:2000, CST, #3580 S, USA), Oct4 (1:2000, CST, #2750 S, USA), Flag (1:2000, Affinity #T0003, USA), Ubiquitin (1:2000, CST, #3936, USA), β-actin (1:5000, Sigma, #AF441, USA) at 4 °C overnight and then incubated with respective second antibodies. The membranes were developed with the ECL System (Beyotime, #P0018S, China).

### MTT assay

A549 and NCI-H820 cells were seeded into 96-well plates at the density of 4000 cells/200 μL/well. After 12 hours, ACT001 was added with the final concentration of 1 μM, 2 μM, 5 μM, 10 μM, 20 μM, 50 μM, and 100 μM. After 48 hours, MTT (Solarbio, #M1025, China) were added into each well and incubated for another 4 hours. Thereafter, the supernatant was discarded and the precipitate was dissolved with 200 μL DMSO (Solarbio, #D8371, China). The absorbance at 570 nm was measured with a microplate reader (Victor Nivo 5 F, PerkinElmer, American).

### Transwell assay

Transwell assay were performed using transwell chambers filters (8-μm pore, BD Falcon). A549 or NCI-H820 cells were resuspended with serum-free DMEM and seeded into the upper chamber at the density of 5 × 10^4^ cells/well/300 μL. The lower chambers contained 700 μL DMEM with 20% FBS as a chemoattractant. After incubated at 37 °C for 24–48 hours, the cells were fixed with 4% paraformaldehyde for 30 mins and stained with crystal viol (Beyotime, #C0121, China) for another 30 mins at 37 °C. The cells in the upper chamber were removed by a cotton swab and the cells migrated to the lower surface were counted.

### Tumorsphere formation assay

Transfected cells or ACT001-treated cells were harvested and counted. 2000 cells were seeded into 24-well ultralow attachment plates. After 7–10 days, tumorsphere formation efficiency was evaluated.

### Probe pull down assay

A549 and NCI-H820 cell protein lysates were incubated with biotin-coupled ACT001 probe at 3 μM, 10 μM, 30 μM and 100 μM for 2 hours at room temperature. Then, pre-chilled methanol was added and incubated at −20 °C for 30 mins to precipitate the protein. After centrifuged at 14,000 × *g* for 15 mins, the protein was dissolved and incubated with streptavidin conjunct agarose A + G beads at 4 °C overnight. After washed with PBS five times, the binding proteins were detected by western blot assay.

### Luciferase reporter assay

Cells were seeded into 24-well plates and co-transfected with pRL-TK and CD133 promoter plasmid (pGL3-basic-CD133). Then, a dual luciferase-reporter assay was used to quantify luciferase activity according to the manufacturer’s instructions.

### Chromatin immunoprecipitation assay

ChIP assay was performed using Chromatin immunoprecipitation assay kit (Millipore #17-295, USA). The primer designed by Olig2-binding sites was shown as follows: forward 5′-GTGCCTTGAGTGAATGACCCC-CTTG-3′ and reverse 5′-AGCAGCAACAGGGAGCCGAGTACGA-3′. Briefly, pCMV-N-Flag-Olig2 plasmid transfected A549 and NCI-H820 cells were fixed with formaldehyde. Then, the cells were collected and suspended with SDS lysis buffer. After sonication, Olig2 protein was pulled down with anti-Flag antibody or control Rabbit IgG (Beyotime, #A7016, China). The specific DNA fragment binding to Olig2 were detected by PCR and analyzed by agarose gel electrophoresis.

### Immunoprecipitation assay

pCMV-N-Flag-Olig2 plasmid transfected A549 and NCI-H820 cells were lysed in RIPA buffer. Subsequently, the protein lysates were incubated with Flag antibody and protein A + G agarose beads (BIO-RAD, #161-4013 & #161-4023, USA) overnight at 4 °C. Then the mixture was centrifuged at 3000 rpm for 30 s and washed with pre-chilled PBS three times. Finally, binding proteins were boiled and subjected to western blot assay.

### Animal assay

6–8 weeks-old female BAL B/c nude mice were purchased from Beijing HFK Bioscience Co., Ltd. (China). 5 × 10^6^/100 μL NCI-H820 cells were injected into right axilla. When the tumor volume reached 500 mm^3^, the tumor was cut into small pieces (1 × 1 × 1 mm^3^) and then the pieces were transplanted into the right flank of live mice. When tumors reached 50–100 mm^3^, the nude mice were grouped randomly based on the order of retrieval from cages. Cages were housed in random order on shelves. For ACT001 treatment, xenografted mice were orally administrated with PBS or ACT001 (100 mg/kg) respectively, or intraperitoneally injected with ADR (5 mg/kg) every three days for seven times. For si-NC and si-Olig2 groups, negative control siRNA or Olig2 knock-down siRNA were incubated with Lipo2000 and directly injected into the tumors a total of four times. For pCMV-N-Flag or pCMV-N-Flag-Olig2 group, respective vectors were suspended in Lipo2000 and directly injected into the tumors for a total of four times. To ensure unbiased decision, investigators preparing drug combinations did not administer the drugs. The body weight and tumor volume were recorded. Eventually, the mice were sacrificed and the tumor tissues were excised for histological examination and western blot assay.

### Pulmonary metastasis assay

NCI-H820-luci cells were injected into BAL B/c nude mice through the tail vein. After 7 days, d-luciferin potassium salt was administrated by intraperitoneal injection. The mice were sacrificed and the lungs were removed for bioluminescence imaging. Then the lungs and livers were embedded in paraffin for HE staining.

### Statistical analysis

Statistical analysis was performed using GraphPad Prism software 6.02. All the experiments were performed at least three independent replicates. All values were expressed as the mean ± standard deviation (SD). Statistical analyses were done using Student’s *t* tests for two variables or one-way ANOVA analysis for multiple variables. Pearson’s correlation test was used for correlation analysis. *p* value less than 0.05 was considered to be statistically significant.

## Results

### Olig2 was overexpressed in lung cancer tissues

**Fig. 1:**
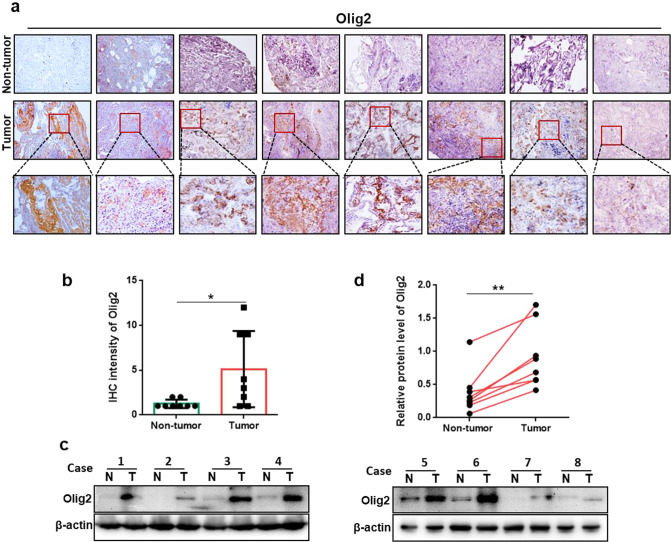
Olig2 was overexpressed in lung cancer tissues. **a** The representative pictures of immunohistochemical staining of Olig2 in paraffin-embedded human lung cancer tissues and non-lung cancer tissues. **b** Statistical analysis of Olig2 immunohistochemical staining of lung cancer tissues and non-lung cancer tissues, *n* = 8. **c** Western blot analysis of Olig2 expression in lung cancer tissues and non-lung cancer tissues, *n* = 8. **d** Statistical analysis of Olig2 expression in lung cancer tissues and non-lung cancer tissues, values were normalized to β-actin, *n* = 8. The data were presented as the mean ± SD, *p < 0.05, **p < 0.01.

Olig2 is a marker of oligodendrocyte that is often used to identify astrocytoma and oligodendrocyte tumor. To determine whether Olig2 plays a key role in lung cancer, immunohistochemical staining was performed to detect the expression of Olig2 in lung cancer clinical specimens (Fig. 1a). Statistical result indicated Olig2 was highly expressed in lung cancer tissues compared with that in non-lung cancer tissues (Fig. 1b). Additionally, Olig2 expression in lung specimens was also detected by western blot assay. The results suggested that the protein level of Olig2 was significantly increased in lung cancer relative to non-lung cancer specimens (Fig. 1c, d). Taken together, these results indicated that Olig2 was significantly overexpressed in lung cancer.

### Olig2 promoted lung cancer cell stemness

**Fig. 2:**
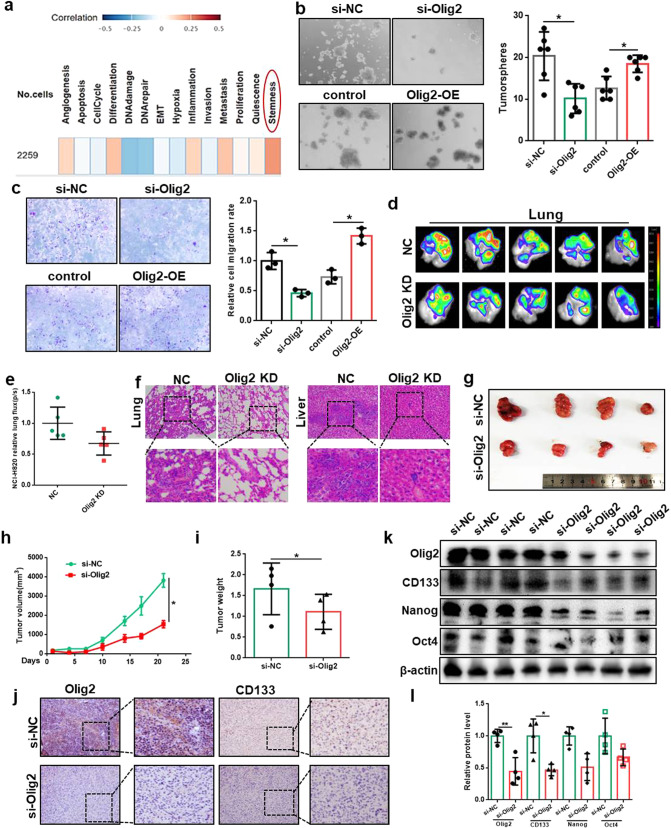
Olig2 promoted lung cancer cell stemness. **a** Average correlations between Olig2 expression and the functional states in glioma. **b** Representative images and number of tumorspheres with Olig2 knockdown or overexpression in NCI-H820 cells, *n* = 6. **c** Representative images and number of NCI-H820 migrated cells with Olig2 knockdown or overexpression, *n* = 3. **d** The bioluminescence imaging of lungs with intravenous injection of NCI-H820-luci cells into BAL B/c nude mice for 7 days, *n* = 5. **e** Statistical analysis of bioluminescence intensity in lungs, *n* = 5. **f** Representative HE staining images of lung and liver metastasis. **g** The picture of xenograft tumors derived from NCI-H820 cells with intra-tumoral injected Olig2 siRNA or si-NC in lipofectamine 2000. **h** Xenograft tumor growth curve of Olig2 siRNA or si-NC-transfected group, *n* = 4. **i** Xenograft tumor weight of Olig2 siRNA or si-NC-transfected group, *n* = 4. **j** Immunohistochemical staining of Olig2 and CD133 of xenograft tumor. **k** Western bolt analysis of Olig2, CD133, Nanog, and Oct4 expression in xenograft tumors. **l** Statistical analysis of Olig2, CD133, Nanog, and Oct4 expression in xenograft tumors, values were normalized to β-actin, *n* = 4. The data were presented as the mean ± SD, *p < 0.05, **p < 0.01.

The functional states of Olig2 in glioma was analyzed by CancerSEA (http://biocc.hrbmu.edu.cn/CancerSEA/). As the result, Olig2 was significantly correlation with stemness in glioma (Fig. 2a). On the basis of this analysis, we wondered whether Olig2 could regulate lung cancer stemness. Cancer cell stemness was charactered by high ability of metastasis and self-renewal. In order to determine the effects of Olig2 on lung cancer stemness, transwell assay, and tumorsphere formation assay were performed after Olig2 knockdown or overexpression. The results indicated that Olig2 knockdown significantly inhibited A549 and NCI-H820 cell migration and tumorsphere formation while Olig2 overexpression clearly increased A549 and NCI-H820 migration and tumorsphere formation (Fig. 2b, c) which suggested Olig2 promoted lung cancer stemness.

Furthermore, we used pulmonary metastasis model and subcutaneous xenografts model to evaluate the effect of Olig2 on lung cancer cell stemness in vivo. The results showed the bioluminescence in lungs were diminished suggesting reduced pulmonary metastasis intensity after Olig2 knock down (Fig. 2d, e). And the HE staining of lung and liver also indicated reduced metastasis intensity after Olig2 knock down (Fig. 2f). Simultaneously, the subcutaneous xenografts model result indicated that the tumor growth rate, tumor volume and tumor weight were significantly decreased in Olig2 knockdown group versus negative control group (Fig. 2g–i). Moreover, to further reveal the effect of Olig2 on lung cancer stemness properties in vivo, immunohistochemical and western blot assay were performed to detect stemness markers including CD133, Nanog and Oct4 expression. From the result, the expression of stemness markers were obviously decreased after Olig2 knock down (Fig. 2j–l). Therefore, all these results suggested Olig2 promoted lung cancer stemness in vivo.

### Olig2 transcriptional regulated CD133 expression

**Fig. 3:**
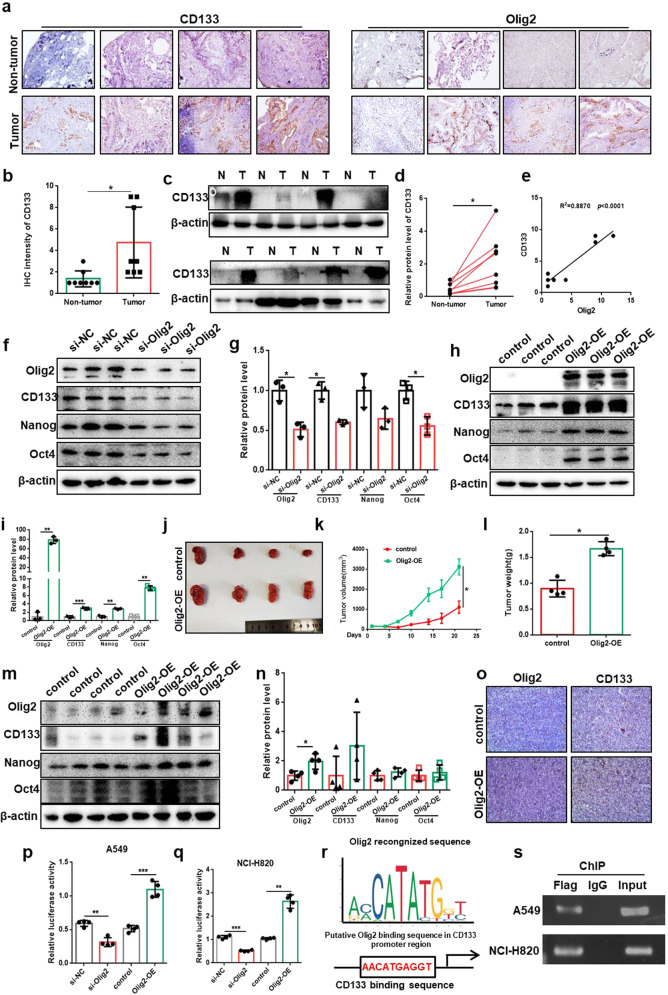
Olig2 transcriptional regulated CD133 expression. **a** The representative pictures of immunohistochemical staining of CD133 and Olig2 in paraffin-embedded human lung cancer tissues and non-lung cancer tissues. **b** Statistical analysis of CD133 immunohistochemical staining of lung cancer tissues and non-lung cancer tissues, *n* = 8. **c** Western blot analysis of CD133 expression in lung cancer tissues and non-lung cancer tissues, *n* = 8. **d** Statistical analysis of CD133 intensity relative to β-actin in lung cancer tissues and non-lung cancer tissues. *n* = 8. **e** Pearson’s correlation analysis of immunohistochemical staining between Olig2 and CD133 expression, *n* = 8. **f** Western blot analysis of Olig2, CD133, Nanog, and Oct4 expression in NCI-H820 cells after Olig2 knockdown with siRNA. **g** Statistical analysis of Olig2, CD133, Nanog, and Oct4 expression in NCI-H820 cells after Olig2 knockdown with siRNA, values were normalized to β-actin. **h** Western blot analysis of Olig2, CD133, Nanog, and Oct4 expression in NCI-H820 cells after Olig2 overexpression with pCMV-N-Flag (control) or pCMV-N-Flag-Olig2 (Olig2-OE) plasmid. **i** Statistical analysis of Olig2, CD133, Nanog and Oct4 expression in NCI-H820 cells after Olig2 overexpression with pCMV-N-Flag (control) or pCMV-N-Flag-Olig2 (Olig2-OE) plasmid, values were normalized to β-actin. **j** The picture of xenograft tumors derived from NCI-H820 cells with intra-tumoral injected pCMV-N-Flag or pCMV-N-Flag-Olig2 in lipofectamine 2000. **k** Xenograft tumor growth curve of pCMV-N-Flag or pCMV-N-Flag-Olig2 transfected group, *n* = 4. **l** Xenograft tumor weight of pCMV-N-Flag or pCMV-N-Flag-Olig2 transfected group, *n* = 4. **m** Western bolt analysis of Olig2, CD133, Nanog, and Oct4 expression in xenograft tumors. **n** Statistical analysis of Olig2, CD133, Nanog, and Oct4 expression in xenograft tumors, values were normalized to β-actin, *n* = 4. **o** Immunohistochemical staining of Olig2 and CD133 of xenograft tumor from pCMV-N-Flag or pCMV-N-Flag-Olig2 transfected group. **p**, **q** CD133 promoter activity by dual-luciferase reporter assays after Olig2 knockdown with siRNA or overexpression with pCMV-N-Flag-Olig2 plasmid in A549 and NCI-H820 cells. **r** Schematic representation of Olig2 binding site to CD133 promoter. **s** The amount of DNA precipitated by either the anti-Flag antibody or control IgG from A549 and NCI-H820 cells. The data were presented as the mean ± SD, *p < 0.05, **p < 0.01, ***p < 0.001.

According to the above preliminary results, stemness marker CD133 level showed positive correlation with Olig2 expression. To confirm this relationship between CD133 and Olig2, we firstly detected the expression of CD133 in eight clinical lung cancer specimens which was used in Fig. 1. The immunohistochemical staining and western blotting results indicated that CD133 was obviously increased in lung cancer tissues compared to non-lung cancer tissues which were consistent with Olig2 expression (Fig. 3a–d). And the correlation analysis appeared to be a strong positive correlation between CD133 and Olig2 (*R*^2^ = 0.8870, Fig. 3e). Furthermore, the expression of CD133 was significantly decreased after Olig2 knock down with siRNA and increased after Olig2 overexpression with pCMV-N-Flag-Olig2 vector which suggested Olig2 promoted lung cancer cell stemness by regulating CD133 expression (Fig. 3f–i). Other stemness markers including Nanog and Oct4 were analyzed simultaneously. The results indicated that Nanog, Oct4, and CD133 were clearly changed with Olig2 expression changed.

Additionally, we implanted NCI-H820 tumor pieces into the right flank of female BAL B/c nude mice, and intra-tumoral injected pCMV-N-Flag or pCMV-N-Flag-Olig2 in lipofectamine 2000 solution. The results on BAL B/c mice indicated that the tumor growth, tumor volume, tumor weight, and CD133 expression was significantly increased in Olig2 overexpression group versus vector control group (Fig. 3j–o). All these findings suggested that Olig2 promoted lung cancer cell stemness and regulated CD133 production. At present, Olig2 was widely reported as a transcription factor [14, 19]. Therefore, we speculated that Olig2 regulate CD133 transcription as a transcriptional factor. To verify whether Olig2 regulates CD133 transcription in lung cancer, we cloned the CD133 promoter sequence into the pGL3 basic luciferase reporter vector and evaluated whether Olig2 could regulate CD133 promoter activity in A549 and NCI-H820 cells. The results indicated Olig2 knockdown resulted in an inhibition of CD133 promoter activity while Olig2 overexpression enhanced the CD133 promoter activity (Fig. 3p, q). Furthermore, according to bioinformatics analyses, a potential Olig2 binding site was found in the promoter region of CD133 (Fig. 3r). To corroborate this speculation, ChIP assay was performed to detect whether Olig2 directly bind the CD133 promoter sequence. After transfected pCMV-N-Flag-Olig2 into A549 and NCI-H820 cells, Flag antibody was used to precipitate the sonicated chromatin from A549 and NCI-H820 cells. The result indicated that after PCR amplification Flag antibody pulled down greater amounts of CD133 promoter DNA than IgG control (Fig. 3s). These data supported the view that Olig2 regulated CD133 expression as a transcription factor by directly binding to the CD133 promoter.

### ACT001 diminished lung cancer cell stemness

**Fig. 4:**
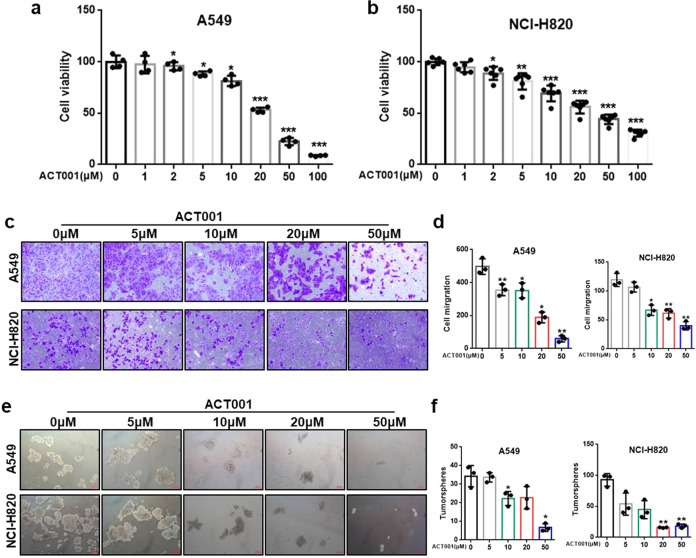
ACT001 diminished lung cancer cell stemness. **a**, **b** The effect of ACT001 on the viability of A549 and NCI-H820 cells for 48 hours. **c**, **d** Representative images and number of A549 and NCI-H820 migrated cells after treatment of ACT001 at indicated concentrations, *n* = 3. **e**, **f** Representative images and number of A549 and NCI-H820 tumorspheres after treatment of ACT001 at indicated concentrations, *n* = 3. The data were presented as the mean ± SD, *p < 0.05, **p < 0.01, ***p < 0.001.

MTT assay was performed to evaluate the effect of ACT001 on lung cancer cell activity. As shown in Fig. 4a, b, ACT001 significantly inhibited proliferation of A549 and NCI-H820 cells in a dose-dependent manner. To further investigate the inhibitory effect of ACT001 on migration of lung cancer cells, the transwell assay of A549 and NCI-H820 cells was performed. The results indicated a marked reduction of lung cancer migrate cells in a dose-dependent manner after ACT001 treatment (Fig. 4c, d). In addition, we assessed the effects of ACT001 on A549 and NCI-H820 cells stemness by tumorsphere formation assay. The result showed that the treatment resulted in a substantial decrease in tumorsphere formation in a dose-dependent manner (Fig. 4e, f). These data indicated that ACT001 was able to effectively reduce lung cancer cell migration and tumorsphere formation to reduce lung cancer stemness.

### ACT001 showed no toxicity and good tolerability for clinical application

In view of the effect of ACT001 on lung cancer stem cells and its future clinical application, we evaluated the toxicology of ACT001. To observe toxic reactions of Beagle dogs, we administered with different doses of ACT001 by gavage for three months. The results showed the trend of body weight gain of dogs in each dose group was consistent with that of control group (Fig. S1a), indicating continuous administration of ACT001 had no apparent toxicity. Whereas the animals experienced dose-dependent decreased spontaneous activity, pronation or lie-down, ataxia, tremor, convulsion, vomiting salivation, hematuria, and other clinical symptoms after receiving the dose of 100 mg/kg and above. While during the recovery period, all the abnormalities recovered which indicated the slight toxicity of ACT001 was reversible. In addition, all treated groups were no obvious degenerations, abnormalities, or lesions found in the major organs. Although individual animals showing physiological fluctuations (Fig. S1b), they can recover spontaneously during the recovery period. This result further confirmed that ACT001 had no obvious toxicity. To investigate whether ACT001 could cause embryotoxicity during embryonic development in rats, we randomly divided pregnant SD rats into five groups: vehicle control group, sodium salicylate group, and different dose of ACT001 groups. The results showed that there was no significant difference in body weight of pregnant rats between ACT001 groups and vehicle control group (Fig. S1c), indicating that ACT001 had no impact on weight gain of pregnant rats. Simultaneously, there was no significant difference in fetal body length, tail length, nest weight, mean fetal weight, or placental weight between ACT001 groups and vehicle control group (Fig. S1d), indicating that ACT001 had no impact on embryo-fetal growth or development. Furthermore, Ames test were conducted to examine whether ACT001 had potential mutagenic effects, the results showed that ACT001 displayed negative characteristics within the concentration range of 0.125 μg/dish~1250 μg/dish in mutagenicity test against strains TA97, TA98, TA100, TA102 and TA1535 with or without S9 (Figs. S1e–f). These data suggested that ACT001 has no physiological toxicity and instruct rational use of drugs in clinic practice.

### ACT001 inhibited Olig2 and CD133 protein level

**Fig. 5:**
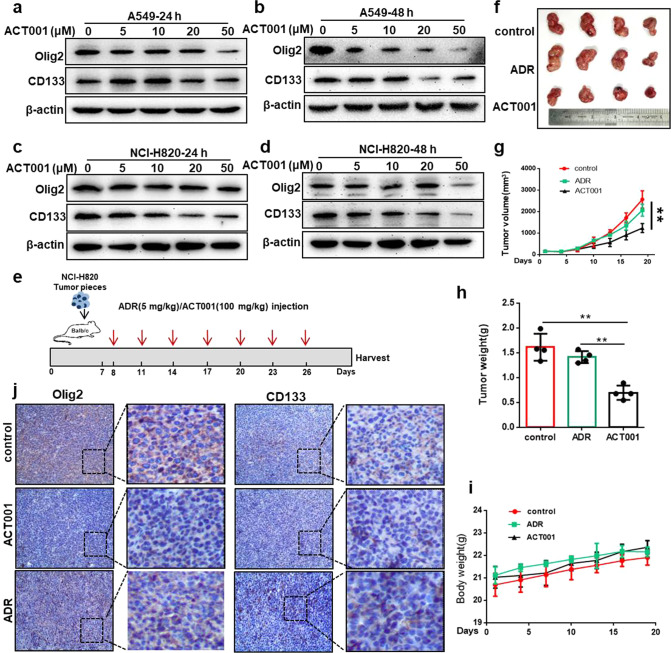
ACT001 inhibited Olig2 and CD133 protein level. **a**, **b** Western blot analyzed Olig2 and CD133 expression in A549 cells after the treatment of ACT001 for 24 or 48 hours at different concentrations. **c**, **d** Western blot analyzed Olig2 and CD133 expression in NCI-H820 cells after the treatment of ACT001 for 24 or 48 hours at different concentrations. **e** Flow chart of drug administration scheme in xenograft tumor model. **f** The picture of xenograft tumors derived from NCI-H820 cells after administration of ACT001 and ADR compared with control group. **g** Xenograft tumor growth curve after ACT001 or ADR administration compared with control group, *n* = 4. **h** Xenograft tumor weight after ACT001 or ADR administration compared with control group, *n* = 4. **i** Body weight after ACT001 or ADR administration compared with control group, *n* = 4. **j** Immunohistochemical staining of Olig2 and CD133 of xenograft tumor after ACT001 or ADR administration compared with control group. The data were presented as the mean ± SD, **p < 0.01.

ACT001 was reported to play a critical role in gliomas by targeting gliomas stem cells through inhibition of AEBP1/PI3K/AKT signaling [21]. Simultaneously, previous results demonstrated Olig2 regulated lung cancer cell stemness by promoting CD133 transcription. Combined all information, we speculated that ACT001 may regulate lung cancer stemness through inhibiting Olig2 level to eventually decrease CD133 expression. To verify this hypothesis, we first treated A549 and NCI-H820 cells with ACT001 and detected Olig2 and CD133 protein expression. The result indicated ACT001 decreased the protein level of Olig2 and CD133 in a dose-dependent manner (Fig. 5a–d).

Furthermore, to examine the effects and mechanism of ACT001 on lung cancer stemness in vivo, we implanted NCI-H820 lung cancer species into right flank of BAL B/c mice. Seven days later, Adriamycin (ADR) was injected with 5 mg/kg by intraperitoneal administration and ACT001 was injected with 100 mg/kg ACT001 by intragastric administration every three days for seven times (Fig. 5e). The results indicated that the tumor volume and tumor weight were decreased significantly after ACT001 administration (Fig. 5f–h), while the body weight did not change obviously in ACT001-treated groups versus control group which suggested the low toxicity and high safety of ACT001 (Fig. 5i). Additionally, we detected the expression of Olig2 and CD133 in tumor tissues using immunohistochemical staining and the results indicated that Olig2 and CD133 protein level was decreased clearly in the ACT001-treated groups versus control group and ADR group (5j). Taken together, all these findings suggested that ACT001 regulates lung cancer stemness by reducing Olig2 levels to eventually decrease CD133 expression. Other stemness markers including Nanog and Oct4 were analyzed simultaneously and the results indicated Nanog and Oct4 was also reduced after ACT001 treatment which suggested ACT001 may have multiple functions (Fig. S2).

### ACT001 regulated CD133 transcription by promoting Olig2 degradation via ubiquitin proteasome pathway

**Fig. 6:**
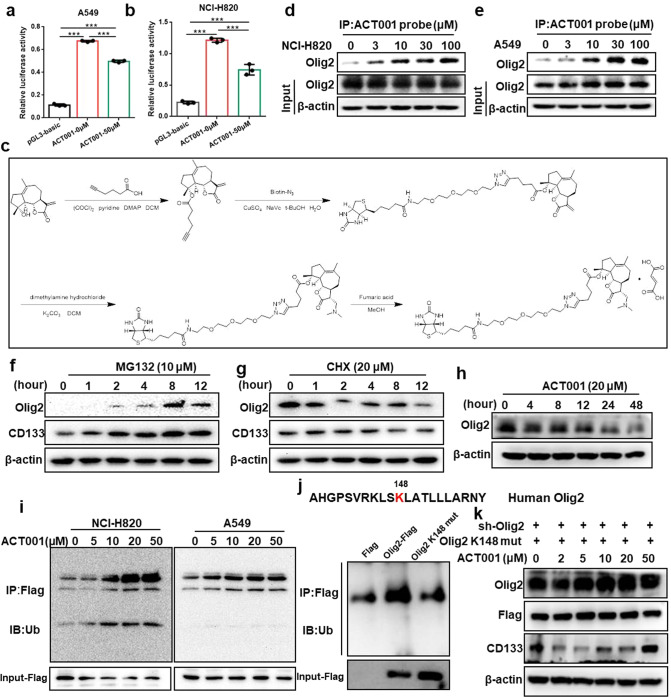
ACT001 regulated CD133 transcription by promoting Olig2 degradation via ubiquitin proteasome pathway. **a**, **b** CD133 promoter activity by dual-luciferase reporter assays after ACT001 treatment for 24 hours in A549 and NCI-H820 cells. **c** Chemical synthesis schematic of ACT001 biotin coupled probe. **d**, **e** A549 and NCI-H820 cell lysates were incubated with ACT001 biotin coupled probe. Then the proteins binding to ACT001 were collected by streptavidin-agarose pull-down and analyzed by western blot assay. **f** Protein level of Olig2 and CD133 in A549 cells treated with MG-132 (10 μM) at indicated time intervals. **g** Protein expression of Olig2 and CD133 in A549 cells treated with cycloheximide (CHX, 20 μM) at indicated time intervals. **h** The kinetics of Olig2 level in NCI-H820 cells after ACT001 treatment at indicated time intervals. **i** The ubiquitylation of Olig2 after treated with ACT001 at different concentrations for 24 hours was determined in A549 and NCI-H820 cells using an anti-Flag antibody after transfected with pCMV-N-Flag-Olig2. **j** The Olig2 ubiquitination sites were predicted by GPS-Uber and verified the ubiquitination level was reduced in K148R mutant cells. **k** CD133 and Olig2 expression were analyzed after treated with ACT001 for 24 hours in K148R mutant cells. The data were presented as the mean ± SD, ***p < 0.001.

We determined the effect of ACT001 in regulating CD133 gene expression through CD133 promoter activity detection. The results indicated that ACT001 treatment apparently inhibited CD133 promoter activity in A549 and NCI-H820 cells (Fig. 6a, b) which demonstrated ACT001 inhibited CD133 transcription. To verify the effect of ACT001 on Olig2 regulation, ACT001 probe was designed and synthesized to pull down the interaction protein with ACT001 (Fig. 6c). After incubated with biotin coupled ACT001 probe, western blot assays were carried out. The result indicated that Olig2 was precipitated by ACT001 probe in a dose-dependent manner (Fig. 6d, e) which indicated that ACT001 directly bound to Olig2. However, ACT001 decreased Olig2 protein level in our previous data. As a result, we deduced ACT001 may influence the stability of Olig2. To confirm our hypothesis, we detected the Olig2 protein level after the treatment of MG132 (a selective 26 S proteasomal inhibitor) or cycloheximide (CHX, protein synthesis inhibitor). The results showed that the Olig2 protein level was remarkably increased with MG-132 treatment in a time-dependent manner (Fig. 6f) while was clearly decreased with CHX treatment in a time-dependent manner (Fig. 6g) in lung cancer cell which suggested that Olig2 protein level could be regulated by ubiquitin proteasome pathway. And the kinetics of Olig2 expression after drug treatment at different times was analyzed which suggested Olig2 protein level obviously changed after ACT001 treatment for 4–8 hours (Fig. 6h).

**Fig. 7:**
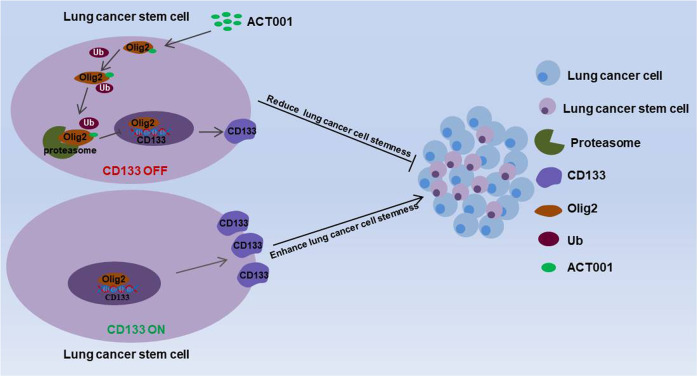
Schematic diagram summarizing the mechanism. ACT001 directly bound to Olig2 protein and promoted Olig2 degradation via ubiquitin-proteasome pathway and eventually regulated lung cancer stemness marker CD133 transcription in lung cancer.

Furthermore, a concomitant increase in Olig2 poly-ubiquitination was analyzed in the presence of ACT001 in both A549 and NCI-H820 cells when Olig2 was immunoprecipitated using a specific antibody (Fig. 6i) which suggested ACT001-induced Olig2 degradation via ubiquitin proteasome pathway. Furthermore, the Olig2 ubiquitination sites were predicted by GPS-Uber (http://gpsuber.biocuckoo.cn/), suggesting that K148 is the potential ubiquitination site. The 148 lysine (K) residue of Olig2 to arginine (R) was mutated and transfected the K148R mutant to Olig2 knockdown cells to construct K148R mutant cell line. The results indicated that the ubiquitination level was reduced in K148R mutant cells (Fig. 6j). Moreover, CD133 and Olig2 expression were analyzed after treated with ACT001 for 24 hours in K148R mutant cells. The results suggested the un-ubiquitin able mutant of Olig2 did not affect CD133 expression after ACT001 treatment in K148R cells (Fig. 6k). Altogether, these results indicated that ACT001 directly bound to Olig2 protein and promoted Olig2 degradation via ubiquitin proteasome pathway and eventually regulate lung cancer stemness marker CD133 transcription in lung cancer (Fig. 7).

## Discussion

There has been the number of molecular alterations identified in lung cancer including oncogenes and tumor suppressor genes, such as Epidermal Growth Factor Receptor (EGFR), Anaplastic Lymphoma Kinase (ALK) which have high relative frequencies as molecular targets [22]. The inhibitor of ALK and EGFR were rapidly developed while the effect in clinical was unsatisfactory. Until now, the overall survival of lung cancer patients has not improved. Lung cancer stem cells (LCSCs) are the root of occurrence, development, recurrence, and drug resistance of lung cancer. The most important characteristics of CSCs are self-renewal capacity and multidirectional differentiation potential to differentiate into ordinary tumor cells [23]. LCSCs maintain their self-renewal ability in a relative quiescent or metabolically inactive state to prevent DNA damage and ROS accumulation which resulting LCSCs tolerated to cell cycle and DNA damage specific agents in cancer therapy [24, 25]. Therefore, new anti-cancer agents and molecular mechanisms which could specific target LCSCs are urgently needed [26]. Currently, one important therapeutic strategy eliminating LCSCs is targeting LCSCs specific signaling pathway including Wnt/β-catenin, Notch pathways, and Hedgehog (Hh) signal pathway [27]. Another important therapeutic strategy eliminating LCSCs is targeting LCSCs characteristic markers including CD133, CD44, CD166, ALDH1, Sox2, Oct4, Nanog, and BMI1 [28, 29]. Until now, vismodegib and sonidegib are only two approved anti-CSCs agents which are Hedgehog inhibitors antagonize the smoothened protein to target CSCs. With the uncertainty efficacy of vismodegib and sonidegib, new targets and anticancer drugs based on new targets are needed.

Olig2 transcription factor is essential for the maintenance of neural progenitor cells. As reported, Olig2 is a potentially highly specific glioma stem cell marker and plays significantly role in reprograming differentiated glioblastoma (GBM) cells into stemlike cells [30]. However, the expression and the mechanism of Olig2 in cancer cell stemness in lung cancer is still unknow. In this article, we discovered that Olig2 was significantly overexpressed in lung cancer, regulated lung cancer metastasis and cancer stemness properties. To discover the mechanism of Olig2 in regulating lung cancer stemness, we found that Olig2 acted as a transcription factor to regulate CD133 expression by directly binding to the CD133 promoter. All these results suggested that Olig2 could be a promising target in anti-LCSCs therapy and new drugs based on Olig2 may exhibit excellent clinical results.

ACT001 showed excellent glioma inhibition in phase I clinical trials and was certified as an orphan drug in the United States and European Union for its prominent effect [31]. And ACT001 exhibited anti-tumor effect by targeting CSCs in various tumors as a multi-target drug. At present, ACT001 exhibited effect in a variety of diseases including glioblastoma [32], breast cancer [33], leukemia [34, 35], and Parkinson’s disease [36]. However, the role of ACT001 in lung cancer was unclear. We further investigated the anti-LCSCs effects and revealed the directly target and the mechanism of ACT001 in lung cancer. The results suggested that ACT001 directly bound to Olig2 protein, induced Olig2 ubiquitination degradation, and inhibit CD133 promoter activity to decrease Olig2 and CD133 protein level in lung cancer. All results indicated that ACT001 may be an outstanding anti-LCSCs drug in lung therapy which provides a reference for expanding the indications of lung cancer in clinical. Moreover, these results conversely verified that Olig2 could be an excellent druggable target in anti-LCSCs therapy which provides a target for further discovery of new anti-LCSCs drugs and therapeutic strategies.

## Supplementary information

Supplementary

## Data Availability

The data used to support the findings of this study are available from the corresponding author upon request.
