# Supplementary material for: ACT001 inhibited CD133 transcription by targeting and inducing Olig2 ubiquitination degradation
Source: Oncogenesis. 2023 Mar 30;12(1):19. doi: 10.1038/s41389-023-00462-6 (PMC10060425; doi:10.1038/s41389-023-00462-6)
Supplement: Supplementary file 1 — Supplementary [file 41389_2023_462_MOESM1_ESM.docx]

**Supplementary methods**

**Long term toxicity in Beagle dogs**

32 Beagle dogs, half male and half female, were selected and randomly divided into control group (Veh), low (L), medium (M) and high (H) dose groups, 8 dogs per group including 4 males and 4 females. Dogs in low, medium and high dose groups were respectively administered with ACT001 at the doses of 40, 100 and 240 mg/kg for 13 continuous weeks (3 months, 6 days per week). And deionized ultrapure water was administrated in control group. To detected the recovery of the toxicity, a 21-day recovery period was set after the last administration. The body weight was recorded and after recovery period, 4 dogs per group, half female and half male, were dissected for histopathological examination.

**Reproductive toxicity assay**

Pregnant SD rats were selected and divided into five groups randomly. Distilled water, sodium salicylate (250 mg/kg) and ACT001 (25 mg/kg, 100 mg/kg and 500 mg/kg) were administered correspondingly according to the groups. On the 6th day of pregnancy (the organogenesis period), ACT001 was administrated by gavage once daily for 10 days. On the 8th day of pregnancy, sodium salicylate was administrated by gavage once daily for 3 days. The general condition of pregnant rats including body weight and growth and development of fetal rats were observed and recorded.

**Ame assay**

Histidine-requiring auxotrophic strain of Salmonella typhimurium was used to detect the mutagenic effect of ACT001. And the effect of ACT001 against TA100 bacterial strain was performed to determine drug dosages. Firstly, 0.1 mL bacterial solution was mixed with 0.1 mL test solution at different concentrations. Then the bacterial solution was divided into metabolic activation group and non-metabolic activation group by adding S9 mixture. The mixture was mixed gently and incubated at 37℃ for 20 min. After the incubation, poured the mixture quickly into the bottom medium, distributed the medium evenly, solidified with horizontal placement, cultured darkly at 37℃ for 72 h and counted revertant colonies per dish. Each experiment was repeated for 3 times.

**Supplementary figure legends**

**Supplementary Figure 1**

ACT001 showed no toxicity and good tolerability for clinical application. (a) The body weight of beagle dogs after ACT001 treatment compared with control group, n=8. (b) H&E staining images of organs which were sacrificed at the 16th week after treatment with ACT001, heart (medium dose group), kindey (high dose group), Bladder (high dose group), liver (high dose group). (c) The body weight of pregnancy rats with ACT001 gavage administration, vehicle group, n=22; positive control, n=23; ACT001-25mg/kg group, n=19; ACT001-100mg/kg, n=23; ACT001-500mg/kg, n=22. (d) Impact on growth and development of fetal rats with ACT001 gavage administration, vehicle group, n=136; positive control, n=112; ACT001-25mg/kg group, n=123; ACT001-100mg/kg, n=103; ACT001-500mg/kg, n=138. (e) Ames test to examine bacterial mutations of ACT001 induced without S9. (f) Ames test to examine bacterial mutations of ACT001 induced with S9. The data are presented as the mean ± SD, **p* <0.05, ***p* <0.05.

**Supplementary Figure 2**

ACT001 inhibited nanog and oct4 expression protein level.(a) Western blot analyzed Nanog and Oct4 expression in NCI-H820 cells after the treatment of ACT001 for 24 h at different concentrations. (b) Western blot analyzed Nanog and Oct4 expression in NCI-H820 cells after the treatment of ACT001 for 48 h at different concentrations.

**Supplementary figure**

**Figure S1 ACT001 showed no toxicity and good tolerability for clinical application**


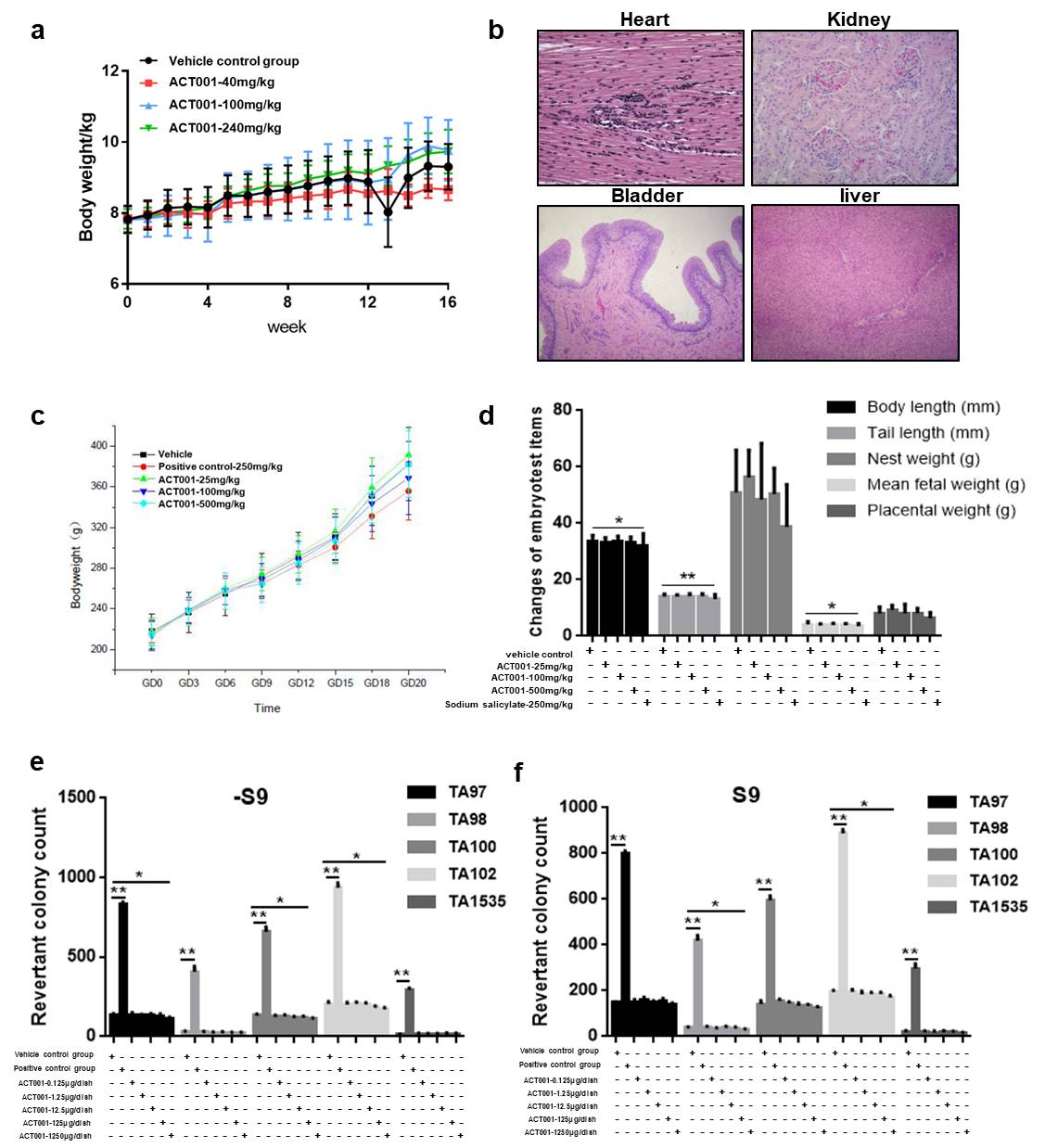


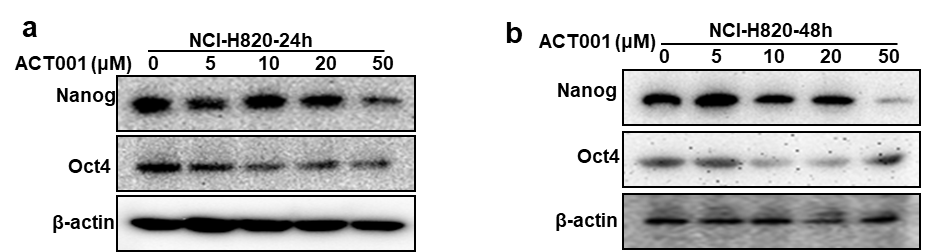
**Figure S2 Nanog and Oct4 expression in NCI-H820 cells after the treatment of ACT001**
